# Weight gain on tenofovir alafenamide fumarate‐based therapy compared to tenofovir disoproxil fumarate‐ and abacavir‐based therapy in children and young people living with HIV in Europe

**DOI:** 10.1111/hiv.70070

**Published:** 2025-07-31

**Authors:** The European Pregnancy and Paediatric Infections Cohort Collaboration (EPPICC) Study Group, Siobhan Crichton, Hannah Castro, Katja Doerholt, Luminita Ene, Luisa Galli, Tessa Goetghebuer, Sannie Brit Nordly, Christoph Königs, Magdalena Marczynska, Andrea Marongiu, Lars Navér, Marisa Navarro, Antoni Noguera‐Julian, Paolo Paioni, Vana Spoulou, Vinicius Vieira, Charlotte Jackson, Intira Jeannie Collins, Ali Judd, Elizabeth Chappell, Laura Calle Miguel, Borja Guarch‐Ibáñez, John O'Rourke, Katia Prime, Beatriz Ruiz‐Saez, Sandra Soeria‐Atmadja, Anna Turkova

**Keywords:** children, HIV, tenofovir alafenamide, treatment, weight, young people

## Abstract

**Objective:**

To compare BMI‐for‐age *z*‐score (zBMI) changes in treatment‐experienced children and young people living with HIV aged 6 to <25 years on tenofovir alafenamide fumarate (TAF) to those on tenofovir disoproxil fumarate (TDF) and abacavir (ABC).

**Methods:**

Cohort study of children and young people living with HIV from 12 European countries were grouped by drug exposure: 1 ‐ on TAF with prior TDF use; 2 ‐ on TAF no prior TDF; 3 ‐ on TDF; and 4 ‐ on ABC. Outcomes, adjusted for characteristics at drug start, were (i) incidence of overweight or obesity by 96 weeks, (ii) zBMI change 48 weeks before/after drug start, (iii) association between TAF/TDF/ABC and anchor drug on zBMI change and (iv) factors associated with zBMI change on TAF over 96 weeks.

**Results:**

Overall, 162, 189, 270 and 144 were in groups 1, 2, 3 and 4, respectively. Median ages at drug start were 16, 13, 14 and 12 years. Obesity incidence by 96 weeks was 16%, 13%, 6% and 12% and higher on TAF than TDF (*p* = 0.018) but comparable to ABC (*p* = 0.568). Similar trends were seen for overweight/obesity, although differences were not significant.

Over 48 weeks, zBMI increased at a higher rate on TAF than TDF (*p* = 0.001) but similar to ABC (*p* = 0.652). zBMI change was higher after than before drug start in group 1 (*p* = 0.059) but similar in other groups.

Over 96 weeks zBMI change on TAF/TDF/ABC combined with dolutegravir vs. other anchor drugs was not statistically different (*p* = 0.196). zBMI change on TAF varied by age (*p* = 0.001), prior TDF (*p* = 0.019), viral load (*p* = 0.042) and zBMI (*p* = 0.004) at TAF start.

**Conclusion:**

zBMI increased on TAF, faster than TDF, but similar to ABC. Weight gain on TAF was associated with multiple factors including prior TDF use.

## INTRODUCTION

Globally, obesity rates are increasing, with 16% of adults and 8% of children and adolescents estimated to be living with obesity in 2022 [1]. Obesity is associated with increased morbidity and mortality from non‐communicable diseases, including cardiovascular disease, diabetes, kidney disease and cancers [2] and childhood obesity is a major risk factor for adulthood obesity [3]. People living with HIV have a higher incidence of comorbidities, including type 2 diabetes, hypertension, chronic kidney disease, hypercholesterolaemia and hypertriglyceridaemia than the general population without HIV, which may be due to the effect of HIV and/or antiretroviral therapy (ART) [4]. There are fewer data in children and young people living with HIV, although the few studies to date have reported similar trends of a high burden of non‐communicable comorbidities [4, 5].

Weight gain among people living with HIV on ART is therefore an increasingly important health concern and has been associated with some specific ART drugs, classes or combinations [6]. Tenofovir alafenamide fumarate (TAF) is a nucleoside reverse transcriptase inhibitor (NRTI), used as an alternative to tenofovir disoproxil fumarate (TDF), due to a better renal and bone safety profile [7]. In some adult studies, increased weight gain has been observed on TAF‐based regimens [8], particularly when combined with dolutegravir (DTG) [8, 9]. Increased weight gain has also been reported after switch from TDF to TAF [10, 11]; however, TDF is known to have a weight suppressing effect which may be driving increases post switch [9].

TAF has been recommended as part of first‐ and second‐line treatment in children and young people living with HIV since 2016 [12, 13, 14], but data on weight changes on TAF in this population are limited. The CHAPAS‐4 trial randomized 919 children and young people living with HIV in Uganda, Zimbabwe and Zambia, aged 3–15 years, and compared TAF‐ and standard of care (abacavir (ABC) or zidovudine (ZDV))‐based second‐line regimens and found greater increases in height‐, weight‐ and body mass index‐for‐age *z*‐scores (zBMI) over 96 weeks on TAF. However, most children had normal or low weight at randomization and the mean increase in weight on TAF was 7.0 kg vs. 6.2 kg on standard of care regimens and was considered as a ‘return to health’ gain [15]. Pooled analysis of four single‐arm trials with 233 children and young people from low‐, middle‐ and high‐income settings aged 6–17 years at time of switch from a suppressive ART regimen to a TAF‐based regimen found zBMI increased by 0.27 over 48 weeks on TAF, and the proportion overweight or obese increased from 13% at TAF start to 23% at 48 weeks [16]. Data from routine care settings from small paediatric observational studies include a Swedish study (*n* = 94) which reported no difference in zBMI at last visit between those on DTG combined with TAF vs. other NRTIs [17]. In a sample of eight treatment‐experienced children in Australia who had a mean zBMI of −0.63 at time of switch to TAF, zBMI increased by 0.61 by 12 months after starting TAF, significantly higher than the −0.02 change observed in the 12 months before TAF start [18].

Our study aimed to describe and compare growth changes and associated factors among children and young people living with HIV on TAF‐, TDF‐ and ABC‐based regimens, in the European Pregnancy and Paediatric Infections Cohort Collaboration (EPPICC).

## METHODS

Individual‐level demographic, ART and clinical data were pooled from 12 observational cohorts in 12 countries across Europe, with access to TAF. Data were pseudonymized and pooled electronically using a modified HIV Cohorts Data Exchange Protocol (HICDEP, www.hicdep.org). EPPICC has ethics committee approval from University College London (reference 17493/001) and all cohorts received approval from local and/or national ethics committees. Cohorts sought informed consent or a waiver of consent in line with national guidelines.

Inclusion criteria for this analysis were age <18 years at HIV diagnosis and ever in paediatric HIV care, age 6 to <25 years and treatment‐experienced (due to small numbers naïve at start of TAF in EPPICC) at start of TAF, TDF or ABC. Among those on TDF or ABC, analysis was restricted to those starting TDF or ABC since 01/01/2013 to provide a comparison group across similar calendar years to TAF while ensuring sufficient sample size. Children and young people who started >1 drug of interest on the same date were excluded. In addition, to ensure changes in zBMI on ABC were not driven by exposure to TDF, children and young people on ABC were excluded if they stopped TDF within 30 days before ABC start or started TDF during the first 96 weeks on ABC.

Those included were categorized into four groups according to TAF, TDF and ABC exposure: group 1 on TAF with prior TDF use (defined as <30 days between TDF discontinuation and TAF start); group 2 on TAF without any prior TDF use; group 3 on TDF; group 4 on ABC. Follow‐up was from drug start until earliest of 25th birthday, dropout, death or last visit, or for those who discontinued TAF/TDF/ABC for >30 days, 7 days after discontinuation. Data following transfer to adult care were included where available. The cut‐off date varied by cohort from 12/2020 to 05/2023.

Height‐for‐age (HAZ), weight‐for‐age (WAZ) and BMI‐for‐age (zBMI) *z*‐scores were calculated using the British 1990 growth reference; stunting was defined as <3rd height percentile, overweight as >85–95th BMI percentile and obesity as >95th BMI percentile [19]. For analyses adjusted for demographic and clinical characteristics, these were sex (male, female), country (United Kingdom/Ireland, Spain, other), ethnic group (Black, White, Other), born outside the country of the cohort (‘born abroad’; yes, no), age at ART initiation and at drug start (6 to <12, 12 to <18, ≥18 years), anchor drug class (DTG, other integrase strand transfer inhibitor (INSTI), protease inhibitor (PI), non‐NRTI (NNRTI), other/mixed), viral load (VL; <50 copies per millilitre (c/mL), ≥50 c/mL, unknown), zBMI and severe immunosuppression‐for‐age [20] (severe, non‐severe, unknown). Analysis was restricted to those with complete demographic and clinical characteristic data, apart from those missing VL or immunosuppression status where unknown categories were included. A window of ±12 weeks was used for HAZ, WAZ, zBMI and immunosuppression at drug start, and −12/+1 weeks for VL.

First, we described characteristics at drug start for each group, and mean change in HAZ, WAZ and zBMI at 24, 48 and 96 (±12) weeks. We then compared zBMI changes on TAF/TDF/ABC by estimating: (i) incidence of new obesity and overweight/obesity by 96 weeks; (ii) BMI‐for‐age *z*‐score (zBMI) change 48 weeks before/after starting TAF/TDF/ABC to assess changes associated with starting a new regimen; (iii) association between TAF/TDF/ABC and DTG use (DTG vs. other anchor) on zBMI change over 96 weeks; and (iv) among those on TAF, factors associated (*p* < 0.1) with zBMI change over 96 weeks. Children and young people with ≥1 zBMI measurement in the first 96 weeks after starting TAF/TDF/ABC were included in analyses (i)–(iv), and for (ii) ≥1 zBMI measurement in both the 48 weeks before and after drug start was also required.

For (i), incidence of new obesity and overweight/obesity was compared across groups using interval‐censored Cox models with clustered standard errors to account for children and young people contributing to >1 drug group. As follow‐up was longer on TDF and ABC, follow‐up was censored at the 90th percentile of TAF follow‐up for all groups. Cox models were adjusted for zBMI and age at TAF/TDF/ABC start only due to low number of events. In (ii)–(iv), mixed models with random time slopes and correlated residuals accounted for repeated measurements. All zBMI measurements from drug start to 96 weeks were included for (i)–(iv), as well as all measurements in the 48 weeks before drug start in (ii) only. Different functions of time and placement of knots in splines were explored and Akaike information criterion was used to select best fitting models. All mixed models were adjusted for demographic and clinical characteristics at drug start and in (iii) characteristics with a significant (*p* < 0.1) interaction with time on TAF added to the model.

In sensitivity analyses, analysis (ii) of changes in zBMI before and after TAF/TDF/ABC start  and analysis (iv) exploring factors associated with growth on TAF only, were repeated (1) in children and young people living with HIV age <18 years at drug start using the WHO growth reference [21], (2) excluding data collected after January 2020 to explore potential indirect impact of the COVID‐19 pandemic, (3) without adjustment for baseline zBMI, thus increasing sample size, and for (ii) only, (4) using propensity score weighting to account for differences between groups.

A summary of analyses is available in Table S1. All analyses were conducted using Stata 18 (StataCorp, College Station, TX, USA).

## RESULTS

In total, 266, 244, 357 and 184 treatment‐experienced children and young people living with HIV were eligible for inclusion in groups 1 (TAF, with prior TDF use), 2 (TAF, no prior TDF), 3 (TDF) and 4 (ABC), respectively; of these, 162 (61%), 189 (78%), 270 (76%) and 144 (78%) had ≥1 BMI measurement in the 96 weeks after starting TAF/TDF/ABC and were included (Table S2). Some children and young people contributed to more than one group; 660 individual children and young people were included, of whom 556 (84%) had eligible time on one drug, 103 (16%) on two and 1 (0.2%) on all three. Adjusted analyses were further restricted to children and young people with complete demographic and clinical data at drug start. Children and young people excluded from analysis due to missing baseline data or no zBMI measurements after drug start were less likely to be Black, born abroad or from the United Kingdom and Ireland and were older at TAF/TDF/ABC start (Table S3).

A similar proportion of children and young people had acquired HIV perinatally (97%, 99%, 96% and 98%) in groups 1–4 and half were born outside the country of the cohort (Table 1). Among those on TAF a higher proportion who had switched from TDF (group 1) were from the United Kingdom and Ireland than those with no prior TDF use (group 2) (56% vs. 35%). Those in group 1 were also older at the start of TAF (median 16 years vs. 13 years), and a lower proportion used DTG as the anchor drug (15% vs. 31%). Children and young people on TDF (group 3) were oldest at ART initiation (median 5 years vs. 3, 2 and 2 years for groups 1, 2 and 4, respectively). Among those on ABC (group 4), a lower proportion were Black (45% vs. 64%, 61% and 61% for groups 1, 2 and 3, respectively), and fewer had previously experienced ART failure (13% vs. 38%, 24% and 34%). Children and young people on TDF and ABC were less likely to have been on an INSTI prior to starting TDF/ABC (1% and 6% in groups 3 and 4 vs. 21% and 22% in groups 1 and 2). The median follow‐up time after drug start was 71 [IQR 34, 136], 99 [45, 165], 120 [58, 194] and 154 [73, 255] weeks for groups 1, 2, 3 and 4, respectively.

**TABLE 1:** Demographic and clinical characteristics of children and young people living with HIV included in the analysis.

	*n* (%) or median [IQR]			
	1: TAF, with prior TDF	2: TAF, no prior TDF	3: TDF	4: ABC
	(*n* = 162)	(*n* = 189)	(*n* = 270)	(*n* = 144)
Female sex	92 (57%)	111 (59%)	148 (55%)	73 (51%)
Ethnicity				
Black	101 (64%)	112 (61%)	163 (61%)	62 (45%)
White	33 (21%)	50 (27%)	61 (23%)	50 (36%)
Other	23 (15%)	23 (12%)	42 (16%)	26 (19%)
Born abroad	78 (49%)	87 (47%)	132 (50%)	67 (50%)
Year of birth				
<2000	37 (23%)	10 (5%)	111 (41%)	21 (15%)
≥2000	125 (77%)	179 (95%)	159 (59%)	123 (85%)
Country				
UK & Ireland	90 (56%)	67 (35%)	157 (58%)	45 (31%)
Spain	46 (28%)	54 (29%)	61 (23%)	48 (33%)
Other	26 (16%)	68 (36%)	52 (19%)	51 (35%)
Perinatally acquired HIV	141 (97%)	167 (99%)	242 (96%)	131 (98%)
Age at ART initiation (years)	3 [0, 9]	2 [0, 7]	5 [1, 9]	2 [0, 6]
**At TAF/TDF/ABC start**				
Age (years)	16 [13, 17]	13 [11, 16]	14 [12, 16]	12 [9, 15]
Age group				
6 to <12 years	24 (15%)	60 (32%)	64 (24%)	71 (49%)
12 to <18 years	105 (65%)	115 (61%)	175 (65%)	64 (44%)
18+ years	33 (20%)	14 (7%)	31 (11%)	9 (6%)
Calendar year	2018 [2017, 2018]	2018 [2017, 2019]	2014 [2013, 2016]	2016 [2014, 2017]
Anchor drug class				
DTG	24 (15%)	58 (31%)	14 (5%)	40 (28%)
Other INSTI	53 (33%)	67 (35%)	9 (3%)	6 (4%)
PI	52 (32%)	45 (24%)	95 (35%)	36 (25%)
NNRTI	21 (13%)	14 (7%)	113 (42%)	49 (34%)
Other/mixed	12 (7%)	5 (3%)	39 (14%)	13 (9%)
Other NRTI in regimen				
FTC	160 (99%)	189 (100%)	202 (75%)	0 (0%)
3TC	0 (0%)	0 (0%)	21 (8%)	134 (93%)
Other	2 (1%)	0 (0%)	47 (17%)	10 (7%)
Anchor drug class in previous regimen				
DTG	19 (12%)	38 (20%)	2 (1%)	5 (3%)
Other INSTI	15 (9%)	4 (2%)	1 (0%)	4 (3%)
PI	68 (42%)	86 (46%)	105 (39%)	59 (41%)
NNRTI	45 (28%)	39 (21%)	102 (38%)	63 (44%)
Other/mixed	15 (9%)	14 (7%)	18 (7%)	6 (4%)
Treatment interruption	0 (0%)	8 (4%)	42 (16%)	7 (5%)
NRTIs in previous regimen				
FTC + TDF	130 (80%)	0 (0%)	0 (0%)	0 (0%)
3TC + ABC	0 (0%)	130 (69%)	113 (42%)	0 (0%)
3TC + ZDV	0 (0%)	27 (14%)	42 (16%)	104 (72%)
Other	32 (20%)	24 (13%)	73 (27%)	33 (23%)
Treatment interruption	0 (0%)	8 (4%)	42 (16%)	7 (5%)
Prior AIDS diagnosis	37 (23%)	40 (21%)	67 (25%)	23 (16%)
Viral load				
<50 c/mL	102 (63%)	107 (57%)	132 (49%)	93 (65%)
≥50 c/mL	36 (22%)	41 (22%)	99 (37%)	23 (16%)
Unknown	24 (15%)	41 (22%)	39 (14%)	28 (19%)
Prior treatment failure	62 (38%)	46 (24%)	93 (34%)	19 (13%)
CD4 count (cells/mm^3^)	657 [430, 942]	721 [560, 1025]	670 [485, 893]	766 [550, 1119]
Severe immunosuppression				
Not severe	110 (68%)	117 (62%)	211 (78%)	104 (72%)
Severe	7 (4%)	5 (3%)	20 (7%)	7 (5%)
Unknown	45 (28%)	67 (35%)	39 (14%)	33 (23%)
Height‐for‐age *z*‐score	−0.44 [−1.04, 0.50]	−0.20 [−0.89, 0.54]	−0.48 [−1.12, 0.49]	−0.19 [−1.08, 0.31]
Stunted	12 (9%)	6 (4%)	25 (11%)	12 (11%)
Weight‐for‐age *z*‐score	0.17 [−0.62, 1.08]	0.11 [−0.61, 0.94]	0.12 [−0.74, 0.95]	−0.06 [−0.92, 0.62]
BMI‐for‐age *z*‐score	0.41 [−0.31, 1.41]	0.29 [−0.48, 1.33]	0.29 [−0.38, 1.26]	0.13 [−0.76, 0.96]
Obese	24 (19%)	24 (16%)	40 (18%)	14 (13%)
Overweight/Obese	45 (35%)	49 (32%)	70 (32%)	27 (24%)

*Note*: Characteristics are summarized among children and young people living with HIV with complete data, except for viral load and immunosuppression status at drug start where an unknown category was included in multivariable analyses. Overall ethnic group was missing for 19 (2%), born abroad status 20 (3%), mode of acquisition 68 (9%), CD4 count at drug start 185(24%), height‐for‐age *z*‐score 157 (21%), weight‐for‐age *z*‐score 129 (17%) and BMI‐for‐age *z*‐score 157 (21%).

Abbreviations: ABC, abacavir; ART, antiretroviral therapy; BMI, body mass index; c/mL, copies/mL; DTG, dolutegravir; FTC, emtricitabine; INSTI, integrase inhibitor; IQR, interquartile range; *n*, number; NRTI, nucleoside reverse transcriptase inhibitor; NNRTI, non‐nucleoside reverse transcriptase inhibitor; PI, protease inhibitor; TAF, tenofovir alafenamide; TDF, tenofovir disoproxil fumarate; 3TC, lamivudine; VL, viral load; UK, United Kingdom; ZDV, zidovudine.

The median HAZ at TAF/TDF/ABC starts below 0 for all groups, and was lowest in those on TAF who had switched from TDF (group 1; −0.44) and those starting TDF (group 3; −0.48) (Table 1). The percentage classed as stunted was 9%, 4%, 11% and 11% in groups 1, 2, 3 and 4, respectively. Median HAZ remained relatively stable over time (Table S4) and very few children and young people newly developed stunting during follow‐up (*n* = 2, 3, 4 and 1 in groups 1, 2, 3 and 4, respectively). Median WAZ at drug start was lowest in those on ABC (group 4; −0.06 vs. 0.17, 0.11, 0.12 for groups 1, 2 and 3, respectively) but increased most in this group (mean change 0.30 vs. 0.12, 0.08 and −0.02 at 96 weeks). Median zBMI at drug start was highest in those on TAF who had switched from TDF (group 1 0.41 vs. 0.29, 0.29 and 0.13 for groups 2, 3 and 4, respectively) with a mean change of 0.20, 0.19, 0.01 and 0.30 in groups 1, 2, 3 and 4, respectively, at 96 weeks.

### Incidence of new overweight and obesity on TAF, TDF and ABC

**FIGURE 1:**
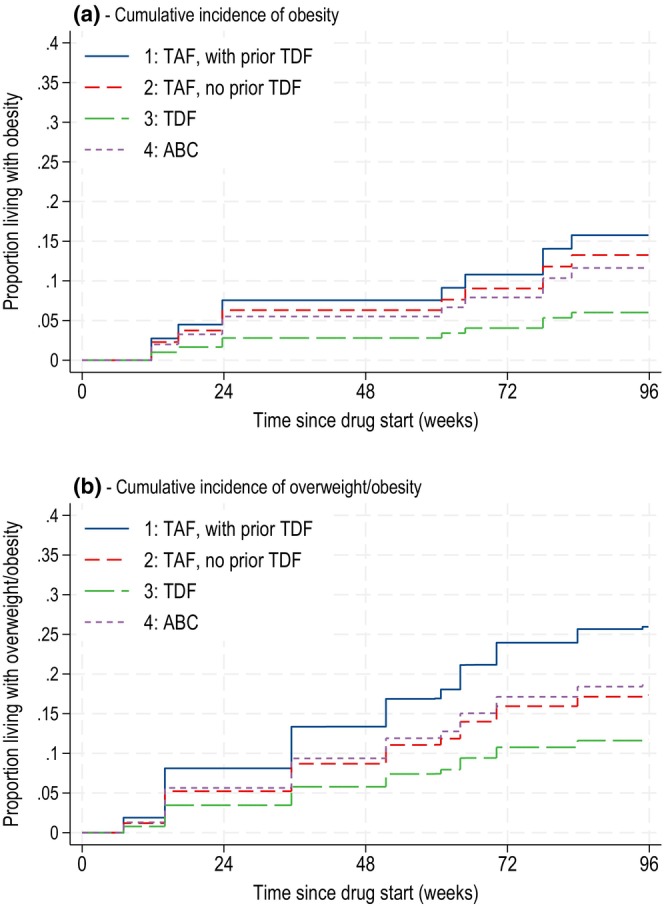
Incidence of overweight and obesity over time on TAF/TDF/ABC. Incidence of new overweight/obesity was estimated among those not living with overweight/obesity at drug start, and incidence of obesity estimated in those not living with obesity at drug start using interval‐censored Cox models. ABC, abacavir; TAF, tenofovir alafenamide; TDF, tenofovir disoproxil fumarate.

At drug start, 19%, 16%, 18% and 13% of groups 1, 2, 3 and 4, respectively, were living with obesity (Table 1). By 96 weeks, the incidence of new obesity was 16%, 13%, 6% and 12%, respectively (Figure 1a), with those on TDF (group 3) least likely to develop obesity (aHR vs. group 2: group 1 1.33 (95% CI 0.61, 2.89); group 3 0.42 (0.20, 0.86); group 4 0.84 (0.46, 1.54)) (Table S5). Similar trends were seen for overweight or obesity with 35%, 32%, 32% and 24% living with overweight/obesity at drug start (Table 1), and 26%, 17%, 12% and 19% newly affected by overweight/obesity by 96 weeks (Figure 1b); however, the difference between groups was not significant (Table S5).

### zBMI changes 48 weeks before and after starting TAF, TDF and ABC

**FIGURE 2:**
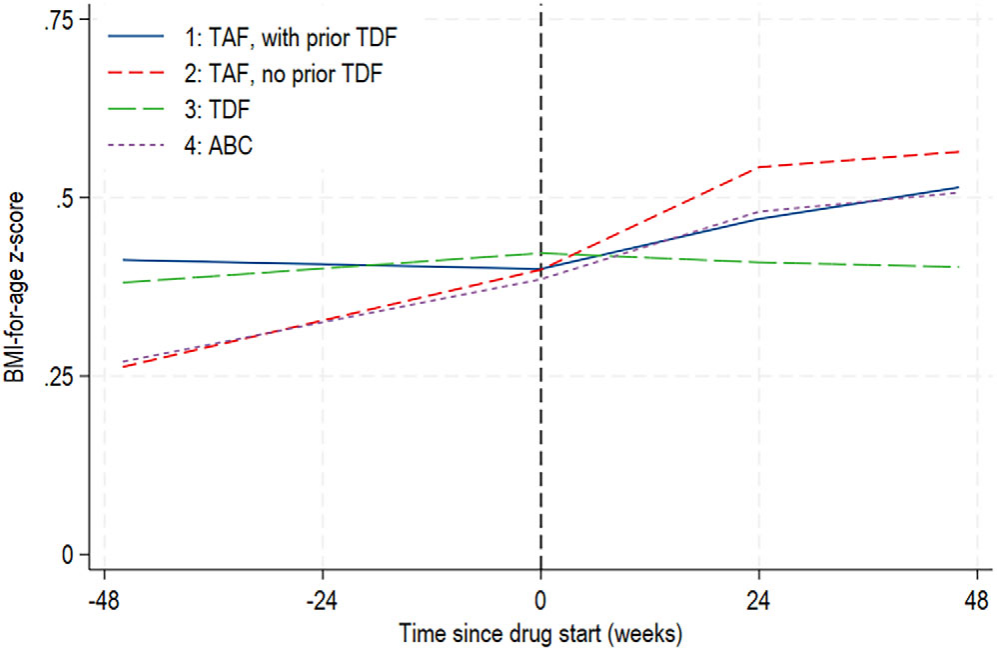
Covariate‐adjusted mean BMI‐for‐age *z*‐score 48 weeks before to 48 weeks after TAF/TDF/ABC start. Model included a linear spline for time. All zBMI measurements from 48 weeks before to 96 weeks after drug start were included, with knots placed at 0 and 24 weeks. Model was adjusted for country group, ethnicity, born abroad, sex, age at ART start, and age, anchor drug class, zBMI, viral load and immunosuppression at drug start. The UK 1990 growth reference was used to derive BMI‐for‐age *z*‐scores. An upward trend in zBMI indicates weight gain greater than the expected rate for age and sex. ABC, abacavir; ART, antiretroviral therapy; BMI, body mass index; CI, confidence interval; TAF, tenofovir alafenamide; TDF, tenofovir disoproxil fumarate; zBMI, BMI‐for‐age *z*‐score.

In groups 1, 2, 3 and 4, 114, 132, 186 and 81 children and young people had data available in the 48 weeks before and after drug start. During the first 48 weeks after drug start, mean zBMI increased for all groups apart from those on TDF (group 3) (Figure 2, Table S6). There was no difference in the mean increase between those on TAF with vs. without prior TDF (group 1 vs. 2; *p* = 0.398), or between the TAF groups and those on ABC (group 1 vs. 2 vs. 4; *p* = 0.652). However, the mean change in zBMI was lower among those on TDF than the TAF groups (group 1 vs. 2 vs. 3; *p* = 0.001). Compared to 48 weeks before drug start, there was a borderline significant difference in the mean zBMI increase in the 48 weeks after drug start among those on TAF who switched from TDF (*p* = 0.059); for other groups, the change in zBMI was similar before and after drug start (*p* = 0.703, 0.253 and 0.971 for groups 2, 3 and 4, respectively).

### Association between DTG use and zBMI change over 96 weeks on TAF, TDF and ABC

**FIGURE 3:**
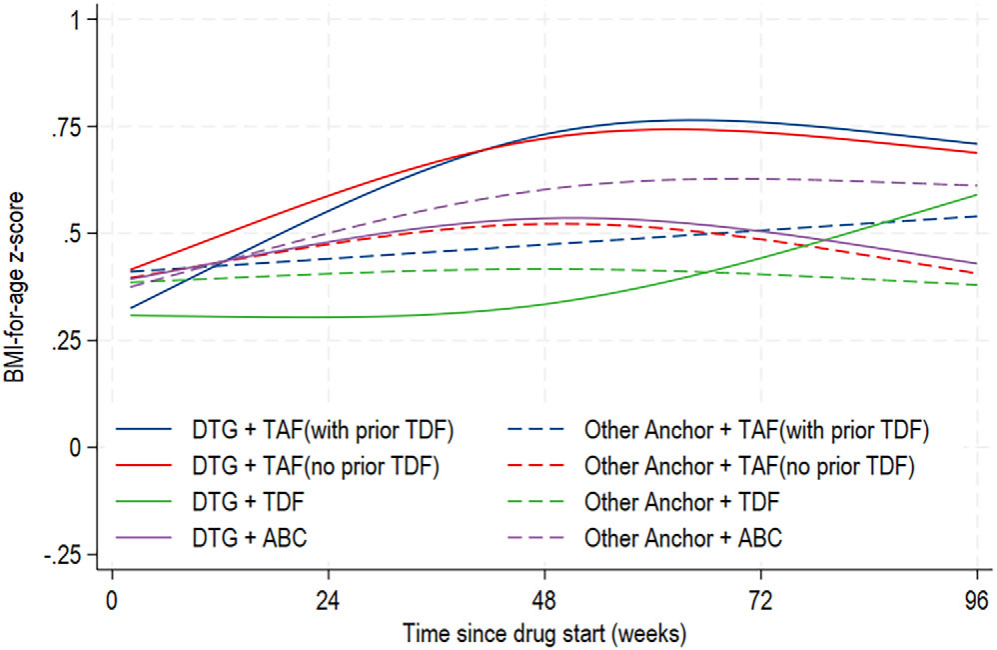
Adjusted mean BMI‐for‐age *z*‐score over 96 weeks on TAF/TDF/ABC by DTG use. Mean zBMI was modelled using a mixed model with a cubic spline for time. Model was adjusted for sex, ethnicity, born abroad, country group, age at ART start, and age, zBMI, viral load and immunosuppression at drug start as main effects and an interaction between NRTI backbone and DTG use. The UK 1990 growth reference was used to derive BMI‐for‐age *z*‐scores. An upward trend in zBMI indicates weight gain greater than the expected rate for age and sex. ABC, abacavir; ART, antiretroviral therapy; BMI, body mass index; DTG, dolutegravir; NRTI, nucleoside reverse transcriptase inhibitor; TAF, tenofovir alafenamide; TDF, tenofovir disoproxil fumarate; zBMI, BMI‐for‐age *z*‐score.

Over 96 weeks, there were slightly greater increases in zBMI among children and young people on DTG + TAF than DTG + TDF or ABC (Figure 3, Table S7) but the interaction between DTG and backbone was not statistically significant (*p* = 0.196).

### Factors associated with zBMI changes over 96 weeks on TAF

**FIGURE 4:**
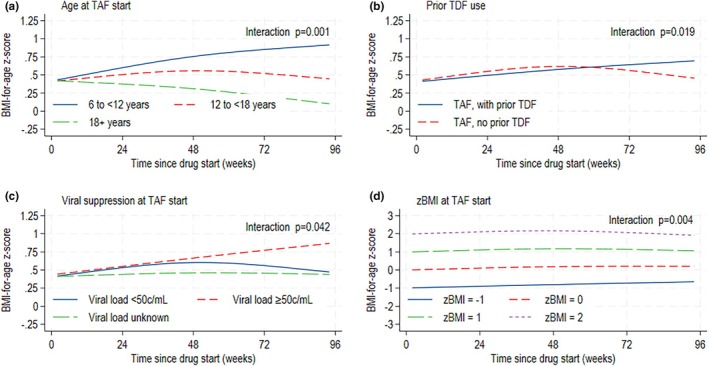
Adjusted mean BMI‐for‐age *z*‐score over 96 weeks on TAF by (a) age, (b) prior TDF use, (c) viral suppression, and (d) zBMI at TAF start. Mean zBMI was modelled using a mixed model with a cubic spline for time. Model was adjusted for sex, ethnicity, born abroad, country group, age at ART start, and age, anchor drug class, zBMI, viral load and immunosuppression at TAF start as main effects, and variables with an interaction with time at the *p* < 0.1 significance level included. The UK 1990 growth reference was used to derive BMI‐for‐age *z*‐scores. An upward trend in zBMI indicates weight gain greater than the expected rate for age and sex. ART, antiretroviral therapy; BMI, body mass index; TAF, tenofovir alafenamide; TDF, tenofovir disoproxil fumarate; zBMI, BMI‐for‐age *z*‐score.

Among children and young people on TAF, the rate of zBMI change differed over time by age (*p* = 0.001), prior TDF use (*p* = 0.019), VL (*p* = 0.042) and zBMI (*p* = 0.004) at TAF start (Figure 4). The greatest increases were in the 6‐ to <12‐year age group, those who switched to TAF from TDF, had unsuppressed HIV VL or lower zBMI at TAF start.

### Sensitivity analysis

In all four sensitivity analyses for changes in zBMI 48 weeks before and after drug start, findings were consistent with the above results (Table S8), including using propensity score weighting where characteristics were well balanced (Table S9). For analyses of characteristics associated with zBMI on TAF, findings were also generally consistent, though sample sizes were smaller and VL at TAF start not significant when baseline zBMI was not adjusted for (*p* = 0.354) or with data censored in 2020 (*p* = 0.141).

## DISCUSSION

To our knowledge, this is the largest study to date assessing changes in weight on TAF in treatment‐experienced children and young people living with HIV in routine care settings. We observed increases in zBMI over 96 weeks after starting TAF, which were most pronounced in children and young people who transitioned from TDF to TAF, were aged 6 to <12 years or were unsuppressed at drug start. However, among children and young people living with HIV without prior TDF exposure, there was an increase in zBMI before starting TAF which continued at a similar rate after TAF start. Just under a fifth of children and young people were living with obesity at the start of TAF; of those who were not, around one in seven developed obesity by 96 weeks.

Over 48 weeks on TAF, zBMI increased by 0.12 in children and young people switching from TDF and 0.17 in those who did not. This is lower than the 0.27 increase reported in a pooled analysis of 223 children and young people living with HIV from Panama, South Africa, Thailand, Uganda and the United States. This may be explained by differences in the populations; the pooled analysis included virally suppressed 6‐ to <18‐year‐olds who had a mean zBMI at the start of TAF of −0.06, and younger age and lower BMI were associated with greater gains in zBMI [16]. While the increases in mean zBMI and incidence of obesity were greater among children and young people on TAF than those on TDF in EPPICC, they were comparable to those on ABC. In contrast, the CHAPAS‐4 trial found increases in zBMI over 96 weeks were 0.10 (95% CI 0.04, 0.16) higher on TAF than standard of care, which included ABC‐containing regimens [15]. However, CHAPAS‐4 participants had lower baseline zBMI (median −1.0[IQR −1.7, −0.4]) with gains in weight probably representing a return to health.

The larger gains observed on TAF and ABC compared to TDF and among those on TAF who switched from TDF have also been observed in adults [11, 22] and point towards a weight suppressive effect of TDF. The mechanisms underlying the suppressive effect of TDF are not fully understood [22]. Some studies suggest TDF may suppress appetite, and TDF has been associated with mitochondrial toxicity and decreased fat mitochondrial DNA which may affect fat distribution [22]. Adult studies have also reported greater weight gain among those on DTG + TAF [8, 9]. In our cohort, there was a trend towards greater weight gain on DTG + TAF than other anchors, but this was not statistically significant.

The broader trends of increases in zBMI in EPPICC among those on ABC and, before TAF start among those not on TDF, indicate increasing zBMI may be associated with other factors and may reflect general population trends. In Europe, 25% of children age <20 years were estimated to be overweight or obese in 2020, and this is forecast to increase to 29% in 2025 [23]. In the United Kingdom, which contributed the most children and young people living with HIV to our analysis, the prevalence of obesity among 10‐ to 11‐year‐olds increased from 18% in 2007/8 to 21% in 2019/20 before a sharp rise to 26% in 2021/22 and then 23% in 2022/23 [24]. As our study included data collected during the COVID‐19 pandemic, during which time hospital appointments were also disrupted in many countries, we conducted sensitivity analyses excluding data collected after the start of 2020 and findings were unchanged.

Around half the children and young people in our analyses were born outside the country of their cohort. Migrants in Europe are often over‐represented in lower socioeconomic groups [25]. Although being born abroad was not associated with zBMI change on TAF, data from the general population in the United Kingdom and Spain suggest growing socioeconomic disparities in childhood obesity. In the United Kingdom, obesity is increasing among 10‐ to 11‐year‐olds in the 10% most deprived neighbourhoods, while remaining stable in the least deprived. In 2007/8, the prevalence of obesity was 10% higher in children in the most deprived neighbourhoods than in the least deprived, and the difference increased to 17% in 2022/23 [24]. Further, in a large cohort of 1.1 million Spanish children aged 2–17 years, there were slight decreases in the prevalence of obesity in the decade to 2016 overall, but increasing socioeconomic disparities [26], and increasing risk of developing overweight/obesity in non‐Spanish nationals [26].

In our EPPICC cohort, the largest increases in zBMI on TAF were in the 6‐ to <12‐year age group. Other European studies have shown that the incidence of childhood obesity in the general population is highest in this age group [24, 26]. We also observed greater increases in zBMI among those who were treatment‐experienced and unsuppressed at the start of TAF. This may reflect a return to health effect and, in adults, greater increases in weight have also been observed after ART initiation in those with higher VLs [6].

This study has several limitations. First, while we adjusted for characteristics at the start of TAF/TDF/ABC, residual confounding may remain. We did not have power to fully adjust for all the individual anchor drugs or other NRTIs, used either before or after starting TAF/TDF/ABC, including drugs such as efavirenz which are also known to have a weight limiting effect. Information on gestational age/birth complication, comorbidities, concomitant medications and psychosocial and lifestyle factors such as physical activity levels, which are known to be associated with growth, was not captured in EPPICC. There were also differences in ethnicity and age at start of TAF/TDF/ABC, and while we adjusted for these factors, biases may arise due to differences in timing of puberty. Children and young people living with HIV often experience delayed pubertal growth spurts which can result in a decline followed by rapid increase in zBMI [28].

Second, a key predictor of change in zBMI over time on a drug is the baseline zBMI at drug start, but 21% of children and young people did not have a baseline measurement available, reducing the sample size in multivariable analyses. However, in sensitivity analyses without baseline BMI our findings were consistent with the main analysis. Third, zBMI was derived using the UK 1990 growth reference which has the advantage of providing a reference up to age 24 years but reflects growth patterns in White children and young people in the United Kingdom and therefore has limitations when applied to our diverse cohort. In sensitivity analyses, using the WHO growth reference (derived from a more diverse population but only includes reference data up to age 19 [21]), we observed similar trends. Finally, the use of BMI has well recognized limitations. While other anthropometric measures such as waist circumference and waist‐to‐hip ratio may more reliably estimates of overweight and obesity [29], these are not routinely captured in clinical practice. The lack of direct body composition data and the ability to distinguish between fat mass or lean mass also limit the interpretation of increases in overall weight.

## CONCLUSION

In conclusion, we found zBMI increased in children and young people living with HIV after starting TAF. For those who transitioned from TDF, weight increased more rapidly after starting TAF than while on TDF, and over 96 weeks, zBMI changes were greater than those without prior TDF use. This suggests discontinuing TDF may contribute to weight gain observed on TAF. For those never on TDF zBMI increases on TAF were similar to growth trends before TAF start and in children and young people living with HIV starting ABC. Together these findings imply weight gain on TAF is multifactorial, influenced in part by prior treatment, and other factors which also influence broader trends in the general population. The potential risk of weight gain and unknown impact on cardiovascular risk must be balanced with the benefits of TAF, such as improved renal function and bone health over TDF.

## AUTHOR CONTRIBUTIONS

All members of the Project team participated in discussions about the study design, choice of statistical analyses and interpretation of the findings and were involved in the preparation and review of the final manuscript. Additionally, Siobhan Crichton and Hannah Castro drafted the manuscript, and Siobhan Crichton, Elizabeth Chappell, Hannah Castro and Charlotte Jackson performed analysis, had access to and verified the data. All members of the Writing group were involved in the collection of data, interpretation of the findings and the preparation and review of the final manuscript.

## FUNDING INFORMATION

This study was funded by Gilead Sciences. Employees of Gilead Sciences are co‐authors of the manuscript and were involved in study design, data interpretation and review of the manuscript. The MRC Clinical Trials Unit at UCL is supported by the Medical Research Council (programme number: MC_UU_00004/03). Other EPPICC activities received industry funding from ViiV Healthcare during the time this work was carried out.

## CONFLICT OF INTEREST STATEMENT

None to declare.

## PATIENT AND PUBLIC INVOLVEMENT

The research was based on secondary data analysis, and there were no direct interactions with patients. This study did not involve patients or members of the public in its design, conduct or dissemination.

## Supporting information


**Data S1.** Supporting Information.

## Data Availability

The EPPICC data are held at MRC CTU at UCL, which encourages optimal use of data by employing a controlled access approach to data sharing, incorporating a transparent and robust system to review requests and provide secure data access consistent with the relevant ethics committee approvals. The rationale for this approach has been published (http://doi.org/10.1186/s13063-015-0604-6). Ethics committee approval for use of EPPICC data restrict the ability for EPPICC data to be shared publicly without request. Rather, ethics approval does allow a controlled access approach. All requests for data are considered and can be initiated by contacting mrcctu.datareleaserequest@ucl.ac.uk.
