# Supplementary material for: Weight gain on tenofovir alafenamide fumarate‐based therapy compared to tenofovir disoproxil fumarate‐ and abacavir‐based therapy in children and young people living with HIV in Europe
Source: HIV Med. 2025 Jul 31;26(9):1418–28. doi: 10.1111/hiv.70070 (PMC12400486; doi:10.1111/hiv.70070)
Supplement: Supplementary file 1 — Data S1. Supporting Information. [file HIV-26-1418-s001.docx]

**Supplementary material**

| **Table S1: Summary of analyses included in study** | | | | |
| --- | --- | --- | --- | --- |
| Analysis | Inclusion criteria | Model | Adjustment | Sensitivity analysis |
| (i) Incidence of overweight/obesity | ≥1 zBMI 96 weeks after start of TAF, TDF, ABC and zBMI <85^th^ centile (i.e. not overweight/obese) at drug start | Interval censored Cox proportional hazards model | Age and zBMI at drug start only (due to low numbers of events) |  |
| (i) Incidence of obesity | ≥1 zBMI 96 weeks after start of TAF, TDF, ABC and zBMI <95^th^ centile (i.e. not obese) at drug start | Interval censored Cox proportional hazards model | Age and zBMI at drug start only (due to low numbers of events) |  |
| (ii) Change in zBMI 48 weeks before and after drug start | ≥1 zBMI 48 weeks before, and ≥1 zBMI 48 weeks after start of TAF, TDF, ABC | Mixed effects model with linear spline for time (knots at 0 and 24 weeks) | Characteristics at ART start/start of drug* as main effects. | 1. zBMI derived using WHO growth reference in CYPLHIV age <18 years at drug start only  2. follow up censored at the start of 2020  3. adjusted for all characteristics at start of drug apart from zBMI  4. propensity score weighted, with weights derived based on all characteristics at drug start |
| (iii) zBMI over 96 weeks on drug by backbone and DTG use | ≥1 zBMI 96 weeks after start of TAF, TDF, ABC | Mixed effects model with cubic spline for time (3 knots) | Characteristics at ART start/start of drug* as main effects. |  |
| (iv) Characteristics associated with zBMI over 96 weeks on TAF | ≥1 zBMI 96 weeks after start of TAF | Mixed effects model with cubic spline for time (3 knots) | Characteristics at ART start/start of drug* as main effects, and interactions between each variable and time added where p<0.1. | 1. zBMI derived using WHO growth reference in CYPLHIV age <18 years at drug start only  2. follow up censored at the start of 2020  3. adjusted for all characteristics at start of drug apart from zBMI |
| *Characteristics included in adjusted analyses were sex, ethnicity, born abroad, country group, age at ART start, and age, anchor drug class, zBMI, viral load and immunosuppression at start of TAF/TDF/ABC  Abbreviations: ABC, abacavir; ART; antiretroviral therapy; CYPLHIV, children and young people living with HIV; DTG, dolutegravir; TAF, Tenofovir alafenamide fumarate; TDF, tenofovir disoproxil fumarate; WHO, World Health Organization; zBMI, body mass index-for-age z-score. | | | | |

| **Table S2: Summary of patient (episodes) eligible for inclusion** | | | | | |
| --- | --- | --- | --- | --- | --- |
|  | 1: TAF, with prior TDF | 2: TAF, no prior TDF | 3: TDF | 4: ABC | Total |
| Total eligible | 266 | 244 | 357 | 184 | 1,051 |
| Included in analysis |  |  |  |  |  |
| ≥1 zBMI on drug | 162 (60.9%) | 189 (77.5%) | 270 (75.6%) | 144 (78.3%) | 765 (72.8%) |
| ≥1 zBMI on drug & complete baseline data | 123 (47.5%) | 149 (63.1%) | 214 (60.1%) | 105 (43.4%) | 591 (54.1%) |
| ≥1 zBMI 48 weeks pre and post | 143 (53.8%) | 165 (67.6%) | 220 (61.6%) | 107 (58.2%) | 635 (60.4%) |
| ≥1 zBMI 48 weeks pre and post & complete baseline data | 114 (45.6%) | 132 (60.3%) | 186 (56.7%) | 81 (37.2%) | 513 (50.5%) |
| Median [IQR] number of zBMI (-48 to +96 weeks) | 5 [4, 8] | 5 [3, 7] | 6 [3, 8] | 6 [3, 8] | 6 [3, 8] |
| Median [IQR] follow-up on drug (weeks) | 71 [34, 136] | 99 [45, 165] | 120 [58, 194] | 154 [73, 255] | 112 [52, 194] |
| This table summarises the numbers of CYPLHIV on each drug who were eligible for inclusion in analysis. CYPLHIV with eligible episodes on >1 study drug were included in each group. Analyses were restricted to those with ≥1 zBMI in the first 96 weeks on drug, apart from models comparing zBMI in the 48 weeks before and after starting TAF/TDF/ABC which required a zBMI in the 48 weeks before and after. Multivariable analyses additionally were restricted to those with complete data on country, ethnicity, born abroad, sex, age at ART initiation, and age, anchor drug class and zBMI at drug start. Overall ethnic group was missing for 19 (2.5%), born abroad status 20 (2.6%) and BMI-for-age z-score 157 (20.5%) who were excluded from analysis. For viral load and immunosuppression at drug start, an unknown category was included in analysis. Abbreviations: ABC, abacavir; CYPLHIV, children and young people living with HIV, IQR, interquartile range; TAF, Tenofovir alafenamide fumarate; TDF, tenofovir disoproxil fumarate; zBMI, body mass index-for-age z-score. | | | | | |

| **Table S3: Demographic and clinical characteristics of CYPLHIV included and excluded from analysis** | | | | |
| --- | --- | --- | --- | --- |
|  | Total | Excluded | Included | p-value |
|  | (n=972) | (n=381) | (n=591) |  |
|  | n(%) or median [IQR] | | | |
| Female sex | 539 (55%) | 206 (54%) | 333 (56%) | 0.486 |
| Ethnicity |  |  |  |  |
| Black | 510 (54%) | 153 (43%) | 357 (60%) | <0.001 |
| White | 291 (31%) | 145 (41%) | 146 (25%) |  |
| Other | 147 (16%) | 59 (17%) | 88 (15%) |  |
| Born abroad | 430 (45%) | 133 (37%) | 297 (50%) | <0.001 |
| Year of birth |  |  |  |  |
| <2000 | 305 (31%) | 171 (45%) | 134 (23%) | <0.001 |
| ≥2000 | 667 (69%) | 210 (55%) | 457 (77%) |  |
| Country |  |  |  |  |
| UK & Ireland | 411 (42%) | 110 (29%) | 301 (51%) | <0.001 |
| Spain | 303 (31%) | 167 (44%) | 136 (23%) |  |
| Other | 258 (27%) | 104 (27%) | 154 (26%) |  |
| Perinatally-acquired HIV | 864 (97%) | 332 (96%) | 532 (98%) | 0.017 |
| Age at ART initiation (years) | 3 [1, 8] | 3 [1, 8] | 3 [1, 8] | 0.640 |
| **At TAF/TDF/ABC start:** | | | | |
| Age (years) | 15 [12, 18] | 17 [13, 20] | 14 [11, 16] | <0.001 |
| Age group |  |  |  |  |
| 6 to <12 years | 242 (25%) | 58 (15%) | 184 (31%) | <0.001 |
| 12 to <18 years | 516 (53%) | 170 (45%) | 346 (59%) |  |
| 18+ years | 214 (22%) | 153 (40%) | 61 (10%) |  |
| Calendar year | 2017 [2015, 2018] | 2017 [2015, 2018] | 2017 [2014, 2018] | 0.641 |
| Anchor drug class |  |  |  |  |
| DTG | 162 (17%) | 48 (13%) | 114 (19%) | <0.001 |
| Other INSTI | 195 (20%) | 96 (25%) | 99 (17%) |  |
| PI | 301 (31%) | 129 (34%) | 172 (29%) |  |
| NNRTI | 225 (23%) | 79 (21%) | 146 (25%) |  |
| Other/mixed | 89 (9%) | 29 (8%) | 60 (10%) |  |
| Other NRTI in regimen |  |  |  |  |
| FTC | 714 (73%) | 282 (74%) | 432 (73%) | 0.719 |
| 3TC | 186 (19%) | 74 (19%) | 112 (19%) |  |
| Other | 72 (7%) | 25 (7%) | 47 (8%) |  |
| Anchor drug class in previous regimen |  |  |  |  |
| DTG | 83 (9%) | 33 (9%) | 50 (8%) | 0.806 |
| Other INSTI | 37 (4%) | 18 (5%) | 19 (3%) |  |
| PI | 391 (40%) | 156 (41%) | 235 (40%) |  |
| NNRTI | 300 (31%) | 110 (29%) | 190 (32%) |  |
| Other/mixed | 73 (8%) | 29 (8%) | 44 (7%) |  |
| Treatment interruption | 88 (9%) | 35 (9%) | 53 (9%) |  |
| NRTIs in previous regimen |  |  |  |  |
| FTC+TDF | 189 (19%) | 94 (25%) | 95 (16%) | 0.001 |
| 3TC+ABC | 282 (29%) | 89 (23%) | 193 (33%) |  |
| 3TC+ZDV | 200 (21%) | 72 (19%) | 128 (22%) |  |
| Other | 213 (22%) | 91 (24%) | 122 (21%) |  |
| Treatment interruption | 88 (9%) | 35 (9%) | 53 (9%) |  |
| Prior AIDS diagnosis | 228 (23%) | 102 (27%) | 126 (21%) | 0.050 |
| Viral load |  |  |  |  |
| <50 c/mL | 528 (54%) | 183 (48%) | 345 (58%) | <0.001 |
| ≥50 c/mL | 260 (27%) | 94 (25%) | 166 (28%) |  |
| Unknown | 184 (19%) | 104 (27%) | 80 (14%) |  |
| Prior treatment failure | 314 (32%) | 152 (40%) | 162 (27%) | <0.001 |
| CD4 count (cells/mm3) | 676 [495, 951] | 661 [490, 951] | 698 [500, 950] | 0.791 |
| Severe Immunosuppression |  |  |  |  |
| Not severe | 662 (68%) | 227 (60%) | 435 (74%) | <0.001 |
| Severe | 51 (5%) | 18 (5%) | 33 (6%) |  |
| Unknown | 259 (27%) | 136 (36%) | 123 (21%) |  |
| Height-for-age z-score | -0.31 [-1.03, 0.46] | -0.03 [-0.90, 1.10] | -0.31 [-1.03, 0.45] | 0.169 |
| Stunted | 56 (9%) | 2 (9%) | 54 (9%) | 0.941 |
| Weight-for-age z-score | 0.10 [-0.72, 0.96] | 0.02 [-1.06, 0.84] | 0.12 [-0.70, 0.98] | 0.193 |
| BMI-for-age z-score | 0.30 [-0.46, 1.26] | 0.36 [-0.70, 1.25] | 0.29 [-0.46, 1.28] | 0.455 |
| Obese | 102 (17%) | 3 (16%) | 99 (17%) | 0.912 |
| Overweight/Obese | 192 (31%) | 5 (26%) | 187 (32%) | 0.623 |
| Characteristics of CYPLHIV with ≥1 zBMI during first 96 weeks on TAF/TDF/ABC and complete data at start of TAF/TDF/ABC are summarised and compared to those who met the overall eligibility data but were excluded due to missing baseline data or having no zBMI available after start of drug. Abbreviations: ABC, abacavir; ART, antiretroviral therapy; BMI, body mass index; c/mL, copies/mL; CYPLHIV, children and young people living with HIV; DTG, dolutegravir; FTC, emtricitabine; INSTI, integrase inhibitor; IQR, interquartile range; n, number; NRTI, nucleoside reverse transcriptase inhibitor; NNRTI, non-nucleoside reverse transcriptase inhibitor; PI, protease inhibitor; TAF, tenofovir alafenamide; TDF, tenofovir disoproxil fumarate; 3TC, lamivudine; VL, viral load; UK, United Kingdom; ZDV, zidovudine. | | | | |

| **Table S4: Change in growth z-scores by duration on drug** | | | | |
| --- | --- | --- | --- | --- |
|  | 1: TAF, with prior TDF | 2: TAF, no prior TDF | 3: TDF | 4: ABC |
|  | median [IQR] or mean (95% CI) | | | |
| HAZ at drug start | -0.44 [-1.04, 0.50] (n=127) | -0.20 [-0.89, 0.54] (n=152) | -0.48 [-1.12, 0.49] (n=219) | -0.19 [-1.08, 0.31] (n=110) |
| Change at 24 weeks | 0.01 (-0.03 0.05) (n=93) | 0.01 (-0.04 0.06) (n=111) | -0.05 (-0.08 -0.02) (n=138) | 0.04 (-0.02 0.10) (n=80) |
| Change at 48 weeks | 0.00 (-0.07 0.08) (n=68) | -0.02 (-0.08 0.05) (n=88) | -0.07 (-0.12 -0.03) (n=149) | 0.10 (0.01 0.19) (n=82) |
| Change at 96 weeks | 0.02 (-0.09 0.14) (n=45) | 0.03 (-0.10 0.17) (n=68) | -0.12 (-0.21 -0.03) (n=108) | 0.14 (0.01 0.26) (n=67) |
| WAZ at drug start | 0.17 [-0.62, 1.08] (n=133) | 0.11 [-0.61, 0.94] (n=161) | 0.12 [-0.74, 0.95] (n=231) | -0.06 [-0.92, 0.62] (n=111) |
| Change at 24 weeks | 0.04 (-0.02 0.11) (n=99) | 0.09 (0.04 0.13) (n=118) | -0.02 (-0.07 0.03) (n=156) | 0.07 (-0.00 0.14) (n=82) |
| Change at 48 weeks | 0.12 (0.02 0.21) (n=74) | 0.10 (0.01 0.19) (n=96) | -0.05 (-0.11 0.01) (n=163) | 0.18 (0.07 0.28) (n=84) |
| Change at 96 weeks | 0.12 (-0.05 0.29) (n=51) | 0.08 (-0.06 0.23) (n=71) | -0.02 (-0.12 0.08) (n=121) | 0.30 (0.13 0.47) (n=68) |
| zBMI at drug start | 0.41 [-0.31, 1.41] (n=127) | 0.29 [-0.48, 1.33] (n=152) | 0.29 [-0.38, 1.26] (n=218) | 0.13 [-0.76, 0.96] (n=111) |
| Change at 24 weeks | 0.05 (-0.03 0.12) (n=93) | 0.11 (0.05 0.17) (n=110) | -0.00 (-0.07 0.07) (n=137) | 0.08 (-0.02 0.18) (n=81) |
| Change at 48 weeks | 0.14 (0.02 0.26) (n=68) | 0.14 (0.02 0.25) (n=87) | -0.02 (-0.10 0.05) (n=148) | 0.19 (0.05 0.33) (n=83) |
| Change at 96 weeks | 0.20 (0.02 0.39) (n=45) | 0.19 (0.03 0.34) (n=67) | 0.01 (-0.10 0.12) (n=108) | 0.30 (0.10 0.51) (n=67) |
| Median z-scores at drug start calculated among those with data available (+/-12 weeks). Mean (95% CI) change in z-scores from drug start estimated among those with data available at 24, 48 or 96 (+/-12) weeks and at drug start. HAZ, WAZ and zBMI were derived using the UK 1990 growth reference. Abbreviations: ABC, abacavir; CI, confidence interval; HAZ, height-for-age z-score; IQR, interquartile range; TAF, tenofovir alafenamide; TDF, tenofovir disoproxil fumarate; WAZ, weight-for-age z-score; zBMI, body mass index-for-age z-score. | | | | |

| **Table S5: Hazard of new obesity and overweight/obesity by drug** | | | | | | |
| --- | --- | --- | --- | --- | --- | --- |
|  | Obesity | | | Overweight/Obesity | | |
|  | HR | 95% CI | p-value | HR | 95% CI | p-value |
| Unadjusted |  |  |  |  |  |  |
| 1: TAF, with prior TDF | 1.21 | 0.54 2.69 | 0.648 | 1.58 | 0.72 3.45 | 0.255 |
| 2: TAF, no prior TDF | 1.00 |  |  | 1.00 |  |  |
| 3: TDF | 0.44 | 0.18 1.07 | 0.070 | 0.66 | 0.31 1.39 | 0.271 |
| 4: ABC | 0.87 | 0.41 1.86 | 0.717 | 1.08 | 0.52 2.23 | 0.831 |
| Adjusted for age and zBMI at drug start |  |  |  |  |  |  |
| 1: TAF, with prior TDF | 1.33 | 0.61 2.89 | 0.479 | 1.49 | 0.50 4.42 | 0.473 |
| 2: TAF, no prior TDF | 1.00 |  |  | 1.00 |  |  |
| 3: TDF | 0.42 | 0.20 0.86 | 0.018 | 0.68 | 0.25 1.84 | 0.449 |
| 4: ABC | 0.84 | 0.46 1.54 | 0.568 | 0.94 | 0.45 1.98 | 0.866 |
| Hazard of overweight/obesity was estimated among those not living with overweight/obesity at drug start, and hazard of obesity estimated in those not living with obesity at drug start using interval censored Cox models. Overweight was defined as having a BMI-for-age z-score between the 85th to <95th percentile and obesity at the 95th percentile or above. BMI-for-age z-scores were derived using the UK 1990 growth reference. Abbreviations: ABC, abacavir; BMI, body mass index; CI, confidence interval; HR, hazard ratio; TAF, tenofovir alafenamide; TDF, tenofovir disoproxil fumarate; UK, United Kingdom. | | | | | | |

| **Table S6: Covariate adjusted mean BMI-for-age z-score 48 weeks before to 48 weeks after TAF/TDF/ABC start** | | | | | |
| --- | --- | --- | --- | --- | --- |
|  | 48 weeks before drug start | | 48 weeks after drug start | | |
|  | Mean change | (95% CI) | Mean change | (95% CI) | p-value |
| 1: TAF, with prior TDF | -0.01 | (-0.12, 0.09) | 0.12 | (0.04, 0.20) | 0.059 |
| 2: TAF, no prior TDF | 0.14 | (0.04, 0.24) | 0.17 | (0.09, 0.24) | 0.703 |
| 3: TDF | 0.04 | (-0.04, 0.13) | -0.02 | (-0.08, 0.04) | 0.253 |
| 4: ABC | 0.12 | (-0.01, 0.26) | 0.12 | (0.03, 0.22) | 0.971 |
| 1 vs. 2 vs. 3 vs. 4 |  |  |  |  | 0.001 |
| 1 vs. 2 vs. 4 |  |  |  |  | 0.652 |
| 1 vs. 2 vs. 3 |  |  |  |  | 0.001 |
| 1 vs. 2 |  |  |  |  | 0.398 |
| Model included a linear spline for time. All zBMI measurements from 48 weeks before to 96 weeks after drug start were included, with knots placed at 0 and 24 weeks. Model was adjusted for country group, ethnicity, born abroad, sex, age at ART start, and age, anchor drug class, zBMI, viral load and immunosuppression at drug start.  Abbreviations: ABC, abacavir; ART, antiretroviral therapy; BMI; body mass index; CI, confidence interval; TAF, tenofovir alafenamide; TDF, tenofovir disoproxil fumarate; zBMI, BMI-for-age z-score. | | | | | |

| **Table S7: Mean BMI-for-age z-score over 96 weeks on TAF/TDF/ABC by DTG use** | | | |
| --- | --- | --- | --- |
|  | Mean change | (95% CI) | p-value |
| DTG + TAF (prior TDF) | 0.55 | (0.09, 1.00) | 0.018 |
| Other Anchor + TAF (prior TDF) | 0.13 | (-0.07, 0.34) | 0.208 |
| DTG + TAF (no TDF) | 0.40 | (0.14, 0.66) | 0.003 |
| Other Anchor + TAF (no TDF) | 0.10 | (-0.12, 0.31) | 0.374 |
| DTG + TDF | 0.21 | (-0.32, 0.74) | 0.441 |
| Other Anchor + TDF | 0.02 | (-0.12, 0.16) | 0.796 |
| DTG + ABC | 0.13 | (-0.23, 0.48) | 0.485 |
| Other Anchor + ABC | 0.32 | (0.11, 0.53) | 0.003 |
| Mean zBMI (UK 1990 growth reference) was modelled using a mixed model with a cubic spline for time. Model was adjusted for sex, ethnicity, born abroad, country group, age at ART start, and age, zBMI, viral load and immunosuppression at drug start as main effects and an interaction between NRTI backbone and DTG use. Abbreviations: ABC, abacavir; ART, antiretroviral therapy; BMI; body mass index; DTG, dolutegravir; NRTI, nucleoside reverse transcriptase inhibitor; TAF, tenofovir alafenamide; TDF, tenofovir disoproxil fumarate; UK, United Kingdom; zBMI, BMI-for-age z-score. | | | |

| **Table S8: Sensitivity analysis for mean change in BMI-for-age z-score 48 weeks before and after starting TAF/TDF/ABC** | | | | | |
| --- | --- | --- | --- | --- | --- |
|  | 48 weeks before drug start | | 48 weeks after drug start | | |
|  | Mean change | (95% CI) | Mean change | (95% CI) | p-value |
| Sensitivity 1 - WHO growth reference |  |  |  |  |  |
| 1: TAF, with prior TDF | 0.00 | (-0.11, 0.11) | 0.13 | (0.05, 0.22) | 0.082 |
| 2: TAF, no prior TDF | 0.12 | (0.02, 0.22) | 0.16 | (0.08, 0.23) | 0.630 |
| 3: TDF | 0.02 | (-0.07, 0.10) | -0.03 | (-0.10, 0.03) | 0.387 |
| 4: ABC | 0.11 | (-0.03, 0.25) | 0.12 | (0.03, 0.22) | 0.887 |
| 1 vs. 2 vs. 3 vs. 4 |  |  |  |  | <0.001 |
| 1 vs. 2 vs. 4 |  |  |  |  | 0.844 |
| 1 vs. 2 vs. 3 |  |  |  |  | <0.001 |
| 1 vs. 2 |  |  |  |  | 0.693 |
|  |  |  |  |  |  |
| Sensitivity 2 - no adjustment for baseline zBMI |  |  |  |  |  |
| 1: TAF, with prior TDF | -0.02 | (-0.12, 0.08) | 0.11 | (0.03, 0.18) | 0.057 |
| 2: TAF, no prior TDF | 0.16 | (0.06, 0.25) | 0.17 | (0.10, 0.24) | 0.828 |
| 3: TDF | 0.06 | (-0.02, 0.14) | -0.02 | (-0.08, 0.04) | 0.149 |
| 4: ABC | 0.10 | (-0.03, 0.23) | 0.14 | (0.05, 0.23) | 0.622 |
| 1 vs. 2 vs. 3 vs. 4 |  |  |  |  | <0.001 |
| 1 vs. 2 vs. 4 |  |  |  |  | 0.490 |
| 1 vs. 2 vs. 3 |  |  |  |  | <0.001 |
| 1 vs. 2 |  |  |  |  | 0.232 |
|  |  |  |  |  |  |
| Sensitivity 3 - censored in 2020 |  |  |  |  |  |
| 1: TAF, with prior TDF | -0.00 | (-0.11, 0.10) | 0.13 | (0.04, 0.22) | 0.061 |
| 2: TAF, no prior TDF | 0.11 | (0.00, 0.22) | 0.20 | (0.10, 0.29) | 0.246 |
| 3: TDF | 0.05 | (-0.04, 0.13) | -0.02 | (-0.08, 0.05) | 0.232 |
| 4: ABC | 0.12 | (-0.02, 0.26) | 0.11 | (0.01, 0.21) | 0.920 |
| 1 vs. 2 vs. 3 vs. 4 |  |  |  |  | <0.001 |
| 1 vs. 2 vs. 4 |  |  |  |  | 0.413 |
| 1 vs. 2 vs. 3 |  |  |  |  | <0.001 |
| 1 vs. 2 |  |  |  |  | 0.307 |
|  |  |  |  |  |  |
| Sensitivity 4 - propensity score weighted |  |  |  |  |  |
| 1: TAF, with prior TDF | -0.13 | (-0.34, 0.07) | 0.24 | (0.05, 0.44) | 0.043 |
| 2: TAF, no prior TDF | 0.14 | (-0.05, 0.33) | 0.14 | (0.05, 0.24) | 0.960 |
| 3: TDF | -0.05 | (-0.23, 0.14) | -0.01 | (-0.16, 0.15) | 0.781 |
| 4: ABC | 0.01 | (-0.16, 0.19) | 0.13 | (-0.01, 0.27) | 0.335 |
| 1 vs. 2 vs. 3 vs. 4 |  |  |  |  | 0.228 |
| 1 vs. 2 vs. 4 |  |  |  |  | 0.605 |
| 1 vs. 2 vs. 3 |  |  |  |  | 0.115 |
| 1 vs. 2 |  |  |  |  | 0.364 |
| All zBMI measurements from 48 weeks before to 96 weeks after drug start were included, with knots placed at 0 and 24 weeks. Models were adjusted for country group, ethnicity, born abroad, sex, age at ART start, and age, anchor drug class, zBMI, viral load and immunosuppression at drug start. Abbreviations: ABC, abacavir; ART, antiretroviral therapy; BMI; body mass index; CI, confidence interval; TAF, tenofovir alafenamide; TDF, tenofovir disoproxil fumarate; WHO, World Health Organization; zBMI, BMI-for-age z-score. | | | | | |

| **Table S9: Sensitivity analysis (propensity score weighting) - covariate balance before and after weighting** | | | | | | |
| --- | --- | --- | --- | --- | --- | --- |
|  | B: TAF no TDF | B: TAF no TDF | C: TDF | C: TDF | D: ABC | D: ABC |
|  | Unweighted | Weighted | Unweighted | Weighted | Unweighted | Weighted |
|  | msd | wmsd | msd | wmsd | msd | wmsd |
| UK & Ireland | -0.42 | 0.02 | 0.00 | 0.11 | -0.59 | 0.02 |
| Spain | 0.01 | 0.19 | -0.06 | 0.02 | 0.23 | 0.02 |
| Other country | 0.47 | -0.21 | 0.06 | -0.15 | 0.45 | -0.05 |
| Black ethnicity | -0.03 | -0.04 | -0.11 | -0.03 | -0.53 | -0.04 |
| White ethnicity | 0.07 | 0.03 | 0.06 | -0.08 | 0.44 | -0.00 |
| Other ethnicity | -0.03 | 0.02 | 0.08 | 0.13 | 0.19 | 0.06 |
| Born abroad | -0.00 | -0.23 | -0.04 | -0.01 | -0.15 | -0.14 |
| Female sex | 0.07 | -0.03 | 0.00 | 0.05 | -0.11 | 0.13 |
| Age at ART initiation (years) | -0.22 | -0.08 | 0.04 | 0.09 | -0.21 | -0.10 |
| Age at drug start (years) | -0.54 | 0.35 | -0.35 | 0.05 | -0.72 | 0.09 |
| DTG | 0.51 | -0.23 | -0.20 | -0.10 | 0.49 | -0.19 |
| Other INSTI | 0.02 | -0.00 | -0.74 | 0.08 | -0.72 | -0.04 |
| PI | -0.22 | 0.05 | -0.04 | -0.01 | -0.30 | -0.02 |
| NNRTI | -0.14 | 0.03 | 0.67 | 0.01 | 0.47 | 0.16 |
| Viral load >=50 c/mL | 0.00 | 0.06 | 0.32 | 0.01 | -0.17 | -0.03 |
| Viral load unknown | 0.23 | -0.08 | -0.01 | -0.20 | 0.19 | 0.02 |
| No severe immunosuppression | -0.17 | 0.12 | 0.24 | 0.09 | 0.09 | -0.02 |
| Severe immunosuppression | -0.11 | 0.03 | 0.11 | 0.08 | -0.05 | 0.12 |
| Immunosuppression unknown | 0.24 | -0.14 | -0.32 | -0.14 | -0.07 | -0.04 |
| BMI-for-age z-score at drug start | -0.09 | -0.03 | -0.09 | -0.11 | -0.27 | -0.28 |
| Table shows standardized differences between group A (TAF, with prior TDF) and each of the other groups. Abbreviations: msd, mean standardised difference; wmsd, weighted mean standardised difference. Standardised differences were calculated as difference in unweighted and then weighted means divided by sample standard deviation. wmsd<+/-0.2 was taken to represent adequate balance. Abbreviations: ABC, abacavir; ART, antiretroviral therapy; BMI, body mass index; c/mL, copies/mL; DTG, dolutegravir; INSTI, integrase inhibitor; msd, mean standardised difference; NNRTI, non-nucleoside reverse transcriptase inhibitor; PI, protease inhibitor; TAF, tenofovir alafenamide; TDF, tenofovir disoproxil fumarate; wmsd, weighted mean standardised difference; UK, United Kindgom. | | | | | | |

**Text S1: Collaborating partners and cohorts**

***EPPICC/Penta Coordinating Team***: Elizabeth Chappell, Siobhan Crichton, Intira Jeannie Collins, Giorgia Dalla Valle, Charlotte Duff, Kate Edgar, Carlo Giaquinto, Charlotte Jackson, Ali Judd, Laura Mangiarini, John O’Rourke, Karen Scott, Claire Thorne

***Collaborating cohorts:***

Belgium: Hopital St Pierre Cohort, Brussels: Tessa Goetghebuer, MD, PhD; Marc Hainaut, MD PhD; Wivine Tremerie, Research nurse; Marc Delforge, data manager.

Denmark: Copenhagen, Denmark: Thomas Ulrik Hoffmann, Sannie Brit Nordly

Germany: Frankfurt, Germany: Christoph Konigs

Greece: Greek cohort: Vana Spoulou.

Italy: Infectious Disease Unit, Meyer Children's Hospital, IRCCS, Florence Italy; Department of Health Sciences, University of Florence, Florence, Italy: Luisa Galli, MD; Elena Chiappini, MD, PhD; Catiuscia Lisi, DStat. Infectious Disease Unit, Meyer Children's Hospital, IRCCS, Florence Italy: Carlotta Montagnani, MD PhD; Elisabetta Venturini, MD, PhD.

Poland: Polish paediatric cohort: Head of the team: Prof Magdalena Marczyńska, MD, PhD.  Members of the team: Jolanta Popielska, MD, PhD; Maria Pokorska-Śpiewak, MD, PhD; Agnieszka Ołdakowska, MD, PhD; Konrad Zawadka, MD, PhD; Magdalena Pluta MD, PhD. Administration assistant: Małgorzata Doroba.  Affiliation: Medical University of Warsaw, Poland, Department of Children’s Infectious Diseases; Hospital of Infectious Diseases in Warsaw, Poland.

Romania: "Victor Babes" Hospital Cohort, Bucharest: Dr Luminita Ene.

Spain: CoRISPE-S and Madrid cohort: *Receive funding from:* Estudio del análisis clínico-epidemiológico de la infección por el vih en niños y adolescentes, mujeres embarazadas y sus hijos a nivel nacional. Ministerio Sanidad. Proyect 202007PN0002.

*Paediatrics Units:* María José Mellado, Luis Escosa, Milagros García-López Hortelano, Talía Sainz, Carlos Grasa, Paula Rodríguez (Hospital Universitario La Paz, Madrid); Jose Tomás Ramos, Pablo Rojo, Luis Prieto-Tato, Cristina Epalza, Alfredo Tagarro, Sara Domínguez, Álvaro Ballesteros (Hospital Universitario Doce de Octubre, Madrid); Marta Illán, Arantxa Berzosa, (Hospital Clínico San Carlos, Madrid); Sara Guillén, Beatriz Soto (Hospital Universitario de Getafe, Madrid); María Luisa Navarro, Jesús Saavedra, Mar Santos, Elena Rincón, David Aguilera, Begoña Santiago, Beatriz Lázaro Martín, Andrea López Suárez (Hospital Universitario Gregorio Marañón, Madrid); Amanda Bermejo (Hospital Universitario de Móstoles, Madrid); María Penín (Hospital Universitario Príncipe de Asturias de Alcalá de Henares, Madrid); Jorge Martínez (Hospital Infantil Universitario Niño Jesús, Madrid); Katie Badillo (Hospital Universitario de Torrejón, Madrid); Ana Belén Jiménez (Hospital Fundación Jiménez Díaz, Madrid); Adriana Navas (Hospital Universitario Infanta Leonor, Madrid); Eider Oñate (Hospital Universitario Donostia, Guipúzcoa); Itziar Pocheville (Hospital Universitario Cruces, Vizcaya); Elisa Garrote (Hospital Universitario Basurto, Vizcaya); Elena Colino, Olga Afonso (Hospital Insular Materno Infantil, Gran Canaria); Jorge Gómez Sirvent (Hospital Universitario Virgen de la Candelaria, Tenerife); Mónica Garzón, Vicente Román (Hospital General, Lanzarote); Raquel Angulo (Hospital de Poniente de El Ejido, Almería); Olaf Neth, Lola Falcón (Hospital Universitario Virgen del Rocío, Sevilla); Pedro Terol (Hospital Universitario Virgen de la Macarena, Sevilla); Juan Luis Santos, Álvaro Vázquez (Hospital Universitario Virgen de las Nieves, Granada); Begoña Carazo, Antonio Medina (Hospital Regional Universitario, Málaga); Francisco Lendínez, Mercedes Ibáñez (Complejo Hospitalario Torrecárdenas, Almería); Estrella Peromingo, María Isabel Sánchez (Hospital Universitario Puerta del Mar, Cádiz); Beatriz Ruiz (Hospital Universitario Reina Sofía de Córdoba); Ana Grande (Complejo Hospitalario Universitario Infanta Cristina, Badajoz); Francisco José Romero (Complejo Hospitalario, Cáceres); Carlos Pérez, Alejandra Méndez (Hospital de Cabueñes, Asturias); Laura Calle-Miguel, Virginia Courel del Río (Hospital Universitario Central de Asturias); Marta Pareja (Complejo Hospitalario Universitario, Albacete); Begoña Losada (Hospital Virgen de la Salud, Toledo); Mercedes Herranz,(Hospital Virgen del Camino, Navarra); Matilde Bustillo (Hospital Universitario Miguel Servet, Zaragoza); Pilar Collado (Hospital Clínico Universitario Lozano Blesa, Zaragoza); José Antonio Couceiro (Complejo Hospitalario Universitario, Pontevedra); Leticia Vila (Complejo Hospitalario Universitario, La Coruña); Consuelo Calviño (Hospital Universitario Lucus Augusti, Lugo); Ana Isabel Piqueras, Manuel Oltra (Hospital Universitario La Fe, Valencia); César Gavilán (Hospital Universitario de San Juan de Alicante, Alicante); Elena Montesinos (Hospital General Universitario, Valencia); Marta Dapena (Hospital General, Castellón); Beatriz Jiménez (Hospital Universitario Marqués de Valdecilla, Cantabria); Ana Gloria Andrés (Complejo Hospitalario, León); Víctor Marugán, Carlos Ochoa (Complejo Hospitalario, Zamora); Ana Isabel Menasalvas, Eloísa Cervantes (Hospital Universitario Virgen de la Arrixaca, Murcia) and Paediatric HIV-BioBank integrated in the Spanish AIDS Research Network and collaborating Centers.

*Adults Units:* Cristina Díez, (Hospital Universitario Gregorio Marañón, Madrid). Ignacio Bernardino, María Luisa Montes, Eulalia Valencia, Ana Delgado (Hospital Universitario La Paz, Madrid); Rafael Rubio, Federico Pulido, Otilia Bisbal (Hospital Universitario Doce de Octubre, Madrid); Alfonso Monereo Alonso (Hospital Universitario de Getafe, Madrid); Juan Berenguer, Cristina Díez, Teresa Aldamiz, Francisco Tejerina, Juan Carlos Bernaldo de Quirós, Belén Padilla, Raquel Carrillo, Pedro Montilla, Elena Bermúdez, Maricela Valerio (Hospital Universitario Gregorio Marañón, Madrid); Jose Sanz (Hospital Universitario Príncipe de Asturias de Alcalá de Henares, Madrid); Alejandra Gimeno (Hospital Universitario de Torrejon, Madrid); Miguel Cervero, Rafael Torres (Hospital Universitario Severo Ochoa de Leganés, Madrid); Santiago Moreno, María Jesús Perez, Santos del Campo (Hospital Universitario Ramon y Cajal, Madrid); Pablo Ryan, Jesus Troya (Hospital Universitario Infanta Leonor, Madrid); Jesus Sanz (Hospital Universitario La Princesa, Madrid); Juan Losa, Rafael Gomez (Hospital Universitario Fundacion Alcorcon, Madrid); Miguel Górgolas (Hospital Fundacion Jimenez Diaz, Madrid); Alberto Díaz, Sara de la Fuente (Hospital Universitario Puerta de Hierro de Majadahonda, Madrid); Jose Antonio Iribarren, Marıa Jose Aramburu, Lourdes Martinez (Hospital Universitario Donostia, Guipuzcoa); Ane Josune Goikoetxea (Hospital Universitario Cruces, Vizcaya); Sofia Ibarra, Mireia de la Peña (Hospital Universitario Basurto, Vizcaya); Víctor Asensi (Hospital Universitario Central de Asturias); Michele Hernandez (Hospital Universitario Insular, Gran Canaria); María Remedios Alemán, Ricardo Pelazas, María del Mar Alonso, Ana María López, Dácil García, Jehovana Rodriguez (Hospital Universitario de Canarias, Tenerife); Miguel Angel Cardenes (Hospital Universitario Doctor Negrin, Gran Canaria); Manuel A. Castaño, Francisco Orihuela, Inés Pérez, Mª Isabel Mayorga (Hospital Regional Universitario, Málaga); Luis Fernando Lopez-Cortes, Cristina Roca, Silvia Llaves (Hospital Universitario Virgen del Rocio, Sevilla); Marıa Jose Rios, Jesus Rodriguez, Virginia Palomo (Hospital Universitario Virgen de la Macarena, Sevilla); Juan Pasquau, Coral Garcia (Hospital Universitario Virgen de las Nieves, Granada); Jose Hernandez, Clara Martinez (Hospital Universitario Clinico San Cecilio, Granada); Antonio Rivero, Angela Camacho (Hospital Universitario Reina Sofia, Cordoba); Dolores Merino, Miguel Raffo, Laura Corpa (Hospital Universitario Juan Ramon Jimenez, Huelva); Elisa Martinez, Fernando Mateos, Jose Javier Blanch (Complejo Hospitalario Universitario, Albacete); Miguel Torralba (Hospital Universitario, Guadalajara); Piedad Arazo, Gloria Samperiz (Hospital Universitario Miguel Servet, Zaragoza); Celia Miralles, Antonio Ocampo, Guille Pousada (Hospital Alvaro Cunqueiro, Pontevedra); Alvaro Mena (Complejo Hospitalario Universitario, La Coruna); Marta Montero, Miguel Salavert, (Hospital Universitario La Fe, Valencia); Maria Jose Galindo, Natalia Pretel (Hospital Clinico Universitario, Valencia); Joaquín Portilla, Irene Portilla (Hospital General Universitario, Alicante); Felix Gutierrez, Mar Masia, Cati Robledano, Araceli Adsuar (Hospital General Universitario de Elche, Alicante); Carmen Hinojosa, Begoña Monteagudo (Hospital Clinico, Valladolid); Pablo Bachiller (Hospital General, Segovia); Jesica Abadía (Hospital Universitario Rio Hortega, Valladolid); Carlos Galera, Helena Albendin, Marian Fernandez (Hospital Universitario Virgen de la Arrixaca, Murcia); Jose Ramon Blanco (Complejo Hospitalario San Millan-San Pedro, la Rioja).

Spain: CoRISPE-cat, Catalonia: CoRISPE‐cat receives financial support from the Instituto de Salud Carlos III through the Red Temática de Investigación Cooperativa en Sida (grant numbers RED RIS RD06/0006/0035 yRD06/0006/0021). Members: Hospital Universitari Vall d’Hebron, Barcelona (Pere Soler-Palacín, Maria Antoinette Frick and Santiago Pérez-Hoyos (statistician)), Hospital Universitari del Mar, Barcelona (Núria López), Hospital Universitari Germans Trias i Pujol, Badalona (María Méndez, Clara Carreras), Hospital Universitari JosepTrueta, Girona (Borja Guarch-Ibáñez), Hospital Universitari Arnau de Vilanova, Lleida (Teresa Vallmanya, Laura Minguell-Domingo), Hospital Universitari Joan XXIII, Tarragona (Olga Calavia), Consorci Sanitari del Maresme, Mataró (Lourdes García), Hospital General de Granollers (Maite Coll), Corporació Sanitària Parc Taulí, Sabadell (Valentí Pineda), Hospital Universitari Sant Joan, Reus (Neus Rius), Fundació Althaia, Manresa (Núria Rovira), Hospital Son Espases, Mallorca (Joaquín Dueñas) and Hospital Sant Joan de Déu, Esplugues (Clàudia Fortuny, Anna Gamell, Antoni Noguera-Julian).

Sweden: Karolinska University Hospital, Stockholm, The Swedish InfCareHIV cohort (Lars Navér, Nora Einarsson, Vendela Hagås, Johanna Rubin, Sandra Soeria-Atmadja).

Switzerland: *Members of the Swiss HIV Cohort Study (SHCS) and the Swiss Mother and Child HIV Cohort (MoCHiV) Study:* Abela I, Aebi-Popp K, Anagnostopoulos A, Battegay M, Baumann M, Bernasconi E, Braun DL, Bucher HC, Calmy A, Cavassini M, Ciuffi A, Crisinel PA, Darling K, Dollenmaier G, Duppenthaler A, Egger M, Elzi L, Fehr J, Fellay J, Francini K, Furrer H, Fux CA, Günthard HF (President of the SHCS), Hachfeld A, Haerry D (deputy of "Positive Council"), Hasse B, Hirsch HH, Hoffmann M, Hösli I, Huber M, Jackson-Perry D (patient representatives), Kahlert CR (Chairman of the Mother & Child Substudy), Keiser O, Klimkait T, Kohns M, Kottanattu L, Kouyos RD, Kovari H, Kusejko K (Head of Data Centre), Labhardt N, Martinez de Tejada B, Marzolini C, Metzner KJ, Müller N, Nemeth J, Nicca D, Notter J, Paioni P, Pantaleo G, Perreau M, Polli Ch, Ranieri E, Rauch A (Chairman of the Scientific Board), Salazar-Vizcaya L, Schmid P, Speck R, Stöckle M (Chairman of the Clinical and Laboratory Committee), Tarr P, Thanh Lecompte M, Trkola A, Wagner N, Wandeler G, Weisser M, Yerly S.*Funding:* This study has been financed within the framework of the Swiss HIV Cohort Study, supported by the Swiss National Science Foundation (grant #201369).

UK/Ireland: Collaborative HIV Paediatric Study (CHIPS): CHIPS was funded by the NHS (London Specialised Commissioning Group) and received additional support from Abbott, Boehringer Ingelheim, Bristol-Myers Squibb, GlaxoSmithKline, Gilead Sciences, Janssen and Roche.

CHIPS Steering Committee: Hermione Lyall (chair), Alasdair Bamford, Karina Butler, Katja Doerholt, Conor Doherty, Caroline Foster, Ian Harrison, Julia Kenny, Nigel Klein, Gillian Letting, Paddy McMaster, Fungai Murau, Edith Nsangi, Katia Prime, Andrew Riordan, Fiona Shackley, Delane Shingadia, Sharon Storey, Gareth Tudor-Williams, Anna Turkova, Steve Welch. MRC Clinical Trials Unit: Intira Jeannie Collins, Claire Cook, Siobhan Crichton, Donna Dobson, Keith Fairbrother, Diana M. Gibb, Ali Judd, Marthe Le Prevost, Nadine Van Looy. Integrated Screening Outcome Surveillance Service (ISOSS), UCL: Helen Peters, Kate Francis, Claire Thorne.

Hospitals participating in CHIPS in 2019/20: University Hospitals Birmingham NHS Foundation Trust, Birmingham: L Thrasyvoulou, S Welch; Brighton and Sussex University Hospitals NHS Trust: K Fidler; University Hospitals Bristol NHS Foundation Trust, Bristol: J Bernatoniene, F Manyika; Calderdale and Huddersfield NHS Foundation Trust, Halifax: G Sharpe; Derby Teaching Hospitals NHS Foundation Trust: B Subramaniam; Glasgow Royal Hospital for Children, Glasgow: R Hague, V Price; Great Ormond Street Hospital for Children NHS Foundation Trust, London: J Flynn, N Klein, A Bamford, D Shingadia, K Grant; Oxford University Hospitals NHS Foundation Trust, Oxford: S Yeadon, S Segal; King's College Hospital NHS Foundation Trust, London: S Hawkins; Leeds Teaching Hospitals NHS Trust, Leeds: M Dowie; University Hospitals of Leicester NHS Trust, Leicester: S Bandi, E Percival ; Luton and Dunstable Hospital NHS Foundation Trust, Luton: M Eisenhut; K Duncan; Milton Keynes General University Hospital NHS Foundation Trust, Milton Keynes: L Anguvaa, L Wren, Newcastle upon Tyne Hospitals NHS Foundation Trust, Newcastle: T Flood, A Pickering; The Pennine Acute Hospitals NHS Trust, Manchester: P McMaster C Murphy; North Middlesex University Hospital NHS Trust, London: J Daniels, Y Lees; Northampton General Hospital NHS Trust, Northampton: F Thompson; London North West Healthcare NHS Trust, Middlesex; A Williams, B Williams, S Pope; Barts Health NHS trust, London Dr S Libeschutz; Nottingham University Hospitals NHS Trust, Nottingham: L Cliffe, S Southall; Portsmouth Hospitals NHS Trust, Portsmouth: A Freeman; Raigmore Hospital, Inverness: H Freeman; Royal Belfast Hospital for Sick Children, Belfast: S Christie; Royal Berkshire NHS Foundation Trust, Reading: A Gordon; Royal Children’s Hospital, Aberdeen: D Rosie Hague, L Clarke; Royal Edinburgh Hospital for Sick Children, Edinburgh: L Jones, L Brown; Royal Free NHS Foundation Trust, London: M Greenberg; Alder Hey Children's NHS Foundation Trust, Liverpool: C Benson, A Riordan; Sheffield Children's NHS Foundation Trust, Sheffield: L Ibberson, F Shackley; University Hospital Southampton NHS Foundation Trust, Southampton: S Patel, J Hancock; St George's University Hospitals NHS Foundation Trust, London: K Doerholt, , K Prime, M Sharland, S Storey; Imperial College Healthcare NHS Trust, London: EGH Lyall, C Foster, P Seery, G Tudor-Williams, N Kirkhope, S Raghunanan; Guy's and St Thomas' NHS Foundation Trust, London: Dr Julia Kenny, A Callaghan; University Hospitals of North Midlands NHS Trust, Stoke On Trent: A Bridgwood, P McMaster; University Hospital of Wales, Cardiff: J Evans, E Blake; NHS Frimley Health Foundation Trust, Slough: A Yannoulias.
